# Baltimore Almshouse Infirmary

**Published:** 1830-01

**Authors:** 


### BALTIMORE ALMSHOUSE INFIRMARY.

### *Aortal Aneurism, with Rupture into the Trachea and Œsophagus.* By Thomas H. Wright, M.D.

Henry M'Claskey, age fifty-four, short stature, form muscular and heavy, with marks of great natural strength. The leading symptoms, at the time of admission, were cough, and a constant sense of weight in the chest, increased on exercise, and causing labour of breathing after any considerable effort. The cough was hoarse and dry, without expectoration, not very frequent, not commonly excited or increased by deep breathing; the sense of weight in the chest constant, rather disagreeable than distressing, and not at all impeding lying down or walking about moderately. The patient represented his present symptoms to have come on about three weeks before, previous to which he was, or believed himself to have been, in good health; had been seldom sick, led an active life, and was free from cough or other disorder.

There was no fever. Examined for many days together, the pulse betrayed no sensible fluctuation; it was sixty-five to seven, soft without volume, requiring pressure to distinguish it well, and not resisting with any energy of stroke; it was both a weak and sluggish pulse, though the latter is usually characterised by some force. The general state of the system corresponded with the torpor of the circulation: the man kept his bed, was silent, and seemed indifferent to every thing about him; his usual position supine, countenance dull and drowsy. When asked respecting his state of feeling, complained of annoyance by his cough, and of the sense of weight in his breast; spoke little, rather abruptly, and always in terms implying despondence of getting better.

There were in this case but faint indications for diagnosis,* or prescription. Chronic pulmonary embarrassment was suspected, but of what particular character was uncertain. No leading indication presenting, a palliative course only was prescribed. For many days the patient remained as when admitted. He ate all that was given him; slept great part of every night, and frequently in the day, yet always expressed himself uncomfortable, and denied any improvement in his sensations.

Diarrhœa was prevalent in the infirmary when this man was received; and in a week after his admission he was affected by that disorder, which caused him much inconvenience, and seemed to aggravate his cough; chiefly, perhaps, by causing him to leave his bed frequently, to go to the privy. One morning, in the fourth week of the man's stay in the infirmary, while we were making the usual tour of medical duty through the ward, he came out of the privy closet, (a small apartment at the end of the ward,) and ran toward his bed, coughing very hard. In the next moment the sound of violent vomiting was heard, which caused us to repair instantly to the spot. M'Claskey was lying on the side of his bed, vomiting blood in torrents, and was lifeless in ten seconds.

*Examination.* No extravasated blood in the general cavity of the thorax. The right lung extremely dilated, filling the whole right cavity of the chest, of a deep purple hue, and engorged to the utmost possible degree, not from vascular congestion, but complete injection with blood of all the bronchial passages and cells, to their minutest divisions: no artificial inflation of the lung could possibly have caused a more perfect display of its expansive capacity. The left lung was not at all dilated, and exhibited no unusual colour.

The heart, viewed in situ, gave but a very partial representation of the nature of the lesion; some appearance of a pouch only presenting just beyond the arch of the aorta. The trachea and œsophagus were divided above and brought down, the membranous connexions around the thorax and to the spine separated, and the heart and lungs taken out together. Being now inverted, the state of the parts was readily traceable. At the deepest posterior part of the arch of the aorta, an inch and a half below the root of the left subclavian, was an aneurismal sac, the size of an egg; its parietes

* It proved afterwards that the stethoscope (which I have to acknowledge the error of neglecting to employ in this obscure case) must have plainly revealed, at least, one decided character of lesion in part of the thoracic organs.

soft, and apparently very thin. This was plainly the source of the hemorrhage, and, to trace its communications, the sac was slit open through its greatest length. The coats of the artery (within the limits of the sac) were very thin and tender, and tore rather than cut, when laid open: the sac was empty, except a few delicate layers of soft coagulable lymph. Passing the finger into the sac, it encountered three or four hard, rough, pointed bodies, on each side, within the aneurismal cavity, which were the extremities of three broken rings of the trachea: the points were thin and sharp, as if wasted, and had a roughness, hardness, and brittleness, more of bone than cartilage. The communication with the trachea, and the cause of bloody insufflation of the right lung, were thus explained: it remained to ascertain why the fatal hemorrhage had occurred in the form of a violent and repeated gush by vomiting. When the œsophagus was now detached from the trachea behind down to the borders of the aneurismal sac, it was found to be united by adhesions both with the diseased portion of the trachea and a part of the aneurismal bag. A further separation of the œsophagus from the trachea disclosed an oval opening in the former, large enough to admit the point of a finger, by which the œsophagus communicated with the trachea, just behind where the rings of the latter had given way, which was pretty low on the left side of the trachea. The coats of the œsophagus were very much attenuated over the whole extent of its adhesion to the trachea and sac, and the rent described communicating directly with the current flowing into the trachea after rupture of the rings; the blood seems to have passed freely also by the route to the stomach. Hence its discharge by distinct acts of full vomiting. The stomach contained, after death, about a pound of coagulated blood.

It has been mentioned that the left lung was not dilated, or changed in colour, &c. The cause of this difference in the two lungs was explained while removing the parts from the cavity of the chest. The left lung was found to adhere with great firmness throughout its whole anterior, lateral, and posterior surface, to its own pleura, and by that to the serous membrane of the ribs. The lung was enlarged very much, firm and heavy, and in its whole substance hepatized to so great a degree, that every trace of bronchial tubes and cells was wholly obliterated up to the point where the left bronchus penetrates the lung by its primary branches. From this point there was no channel by which air could enter the lung; and, for that cause, the extravasated blood was totally excluded.

This lung had evidently been long out of the service of breathing, and, as its cohesion and condensation was clearly the work of inflammation, and that probably of no indolent character, it is inexplicable how the unfortunate subject of the case could have been so unconscious as he appeared of any former morbid state of his lungs, and so fully impressed with the belief that his health was perfect till within four weeks of his coming to the Almshouse.

The case, in this respect, adds another to the numberless admonitions, derived from hospital practice especially, how little confidence can be placed in the account which patients give of their general state of health, even where there is no reason to suppose the smallest intention to deceive. It was to the state of the left lung, as represented, that allusion was made, when it was remarked that the stethoscope might have clearly indicated one important feature of diagnosis, in the investigation of the seat, &c. of lesion about the thoracic textures. The respiratory murmur (indeed every sound which respiration gives by mediate auscultation,) was, of course, wholly extinct over the entire front, side, and back surface of the chest, corresponding to the outline and body of the left lung.—*American Journal of Med. Sciences.*

---

